# Patient perceptions of relational continuity in England: insights from two cross-sectional surveys

**DOI:** 10.3399/BJGPO.2024.0267

**Published:** 2025-12-19

**Authors:** Bolanle Odebiyi, Jonathan Gibson, Mhorag Goff, Ali MK Hindi, Jonathan Hammond, Katherine Checkland, Matt Sutton, Sally Jacobs

**Affiliations:** 1 School of Health Sciences, Faculty of Biology, Medicine and Health, The University of Manchester, Manchester, UK; 2 Division of Pharmacy and Optometry, The University of Manchester, Manchester, UK; 3 Health Organisation, Policy and Economics Research Group, The University of Manchester, Manchester, UK; 4 Division of Population Health, Health Services Research & Primary care, The University of Manchester, Manchester, UK

**Keywords:** continuity of patient care, primary health care, general practice

## Abstract

**Background:**

Declining continuity of care in England could disproportionately affect some patient groups.

**Aim:**

To examine patients’ perceptions of continuity of primary care, identify factors associated with continuity, and measure changes in continuity of care for different patient groups, following primary care network (PCN) implementations.

**Design & setting:**

Cross-sectional surveys were undertaken of patients from three groups (older adults with polypharmacy, patients with mild or moderate anxiety or depression, and working-age adults [18–45 years]), attending 19 practices in five PCNs in England, at two timepoints (November 2021–April 2022 and November 2022–April 2023).

**Method:**

Relational continuity was measured using the Nijmegen Continuity Questionnaire (NCQ) score. Differences between patient groups and two timepoints were tested using multiple linear regression.

**Results:**

Survey response rates were *n* = 362/1547 (23%) and *n* = 350/1528 (23%). Adults with polypharmacy experienced significantly better continuity than adults with anxiety or depression and younger working-age adults after adjusting for PCN, practice, and population-level characteristics (*P*< 0.05). Those who always or sometimes saw their preferred GP experienced significantly better continuity than those who never did (*P*< 0.01). PCNs with pre-existing inter-practice collaborations were not associated with differences in relational continuity.

**Conclusion:**

We found differences in experienced continuity of care between patient groups. Relational continuity depends on seeing a preferred GP, not a non-preferred GP or other healthcare professional (HCP). Better continuity was associated with larger practice networks.

## How this fits in

Continuity of care in primary care has been declining in England over recent years. Organisational and workforce developments associated with the introduction of primary care networks (PCNs) may further undermine patients’ experiences of continuity of care. We undertook two cross-sectional surveys to investigate how different groups of patients perceive continuity of care; factors that are associated with continuity of care; and whether continuity has changed over time in response to PCN-related organisational changes. Relational continuity was dependent on seeing a preferred GP compared with a non-preferred GP or other HCP. We found no changes in continuity of care in the second and third years after PCNs were introduced.

## Introduction

Continuity of care is defined here as a patient’s continuing relationship with a healthcare professional (HCP), known as ‘(inter)personal’ or ‘relational’ continuity.^1–5^ It improves patient satisfaction,^6–8^ treatment adherence,^9,10^ and may decrease hospital admissions^11–13^ and mortality.^14^ Relational continuity is valued by general practice HCPs and patients, and considered an indicator of good quality, safe, and efficient primary care^1,3^ in healthcare systems worldwide.^15^ Continuity may be better in some European countries, such as The Netherlands,^16^ with evidence suggesting continuity of care has declined in England.^17–21^

Primary care networks (PCNs) were introduced into the English NHS in 2019 as part of the NHS Long Term Plan.^22^ They bring together groups of neighbouring general practices^23^ to deliver services collaboratively, with funding for shared non-GP ‘additional roles’^24^ to address workforce shortages and improve patients’ access and care quality.^25^ This may result in patients consulting with HCPs other than their preferred individuals, potentially affecting their experience of relational continuity.^26^ PCN-wide care provision may undermine continuity because clinicians dividing their time between member practices may be less available at their ‘base’ practice.^27^

Haggerty *et al* outline the following three dimensions of continuity: relational; management; and informational.^28^ For this study we focused on relational continuity, because there is the most evidence of benefit. While some studies aim to assess relational continuity from medical records,^29,30^ these do not capture patients’ perceptions or experiences. This requires measurement of continuity experiences. While previous studies have linked continuity to organisational characteristics, such as GP numbers, no study has specifically related continuity across patient groups to these characteristics.

This study is the first comprehensive analysis of perceptions of relational continuity of adult patients from different age groups with diverse medical conditions in the PCN context. We examine how continuity has been affected by organisational and workforce changes, asking: (1) How do different patient groups with differing needs for continuity perceive it? (2) What factors were associated with continuity for patients? (3) Has continuity for patients changed over time with PCN-related organisational changes?

## Method

### Design and setting

We used data from cross-sectional surveys conducted at two timepoints: T1 (November 2021–April 2022) and T2 (November 2022–April 2023). Patients who had visited their practice in the preceding 3 months were identified using an EMIS or SystmOne search, excluding those with moderate or severe dementia. A random sample of 25 patients from each patient category was selected. Questionnaires were distributed to these patients by their practices. Two postal reminders were sent out 1 month apart. The study included five PCN areas in England, selected to represent a range of characteristics that may influence continuity experienced by patients, including practice size, area deprivation, and rurality or urbanicity.

### Study population and sample

Three responder categories were selected to represent different experiences and views on continuity of care: (1) older adults with polypharmacy (≥10 medications),^31^ likely to value continuity, be multimorbid,^32^ and have clinical pharmacist appointments for medication reviews; (2) patients with mild or moderate anxiety or depression, whose mental health care is primarily managed within general practice and likely to value continuity; and (3) working-age adults, who may prioritise ease and speed of access over continuity. The sample size was calculated to detect a half-point difference in the mean continuity score (Nijmegen Continuity Questionnaire [NCQ] subscale) between T1 and T2, with an alpha level of 1% and 95% power.

### Primary outcome

The primary outcome was patients' experienced continuity of care,^29,30^ measured using the NCQ. The NCQ assesses continuity as a multidimensional concept across multiple care settings with 28 items divided into two subscales: relational continuity (care provider knows the patient and shows commitment) and team or cross-boundary continuity (collaboration between care providers). Items are rated on a 5-point Likert scale from 'strongly agree' to 'strongly disagree', with an additional 'I do not know' option. The NCQ was adapted to include questions about any HCPs consulted within the previous 3 months and cooperation between HCPs within and outside the practice. Data collected included patient demographics, existing long-term conditions, reason for last visit, HCP seen, and overall experience. Additional questions from the GP Patient Survey provided national data comparison.^33^ Practice or PCN-level organisational variables and area-specific demographic, socioeconomic, and health-needs variables were obtained for each practice.

### Primary outcome measure

The primary outcome was derived from NCQ items constituting the 5-point Likert scale. The NCQ score, a composite measure of continuity experienced with the last HCP (GP, nurse, pharmacist, social prescriber, and/or mental health professional) seen at the general practice was derived from a decision rule (Supplementary Table S1). NCQ items were scored from 1–5 and total subscale scores were calculated as mean item scores from 1–5, where higher scores indicated greater continuity of care experienced. A subscale was calculated as the mean of all items in the subscale, excluding cases with more than one item missing (five in 2021–2022 and 11 in 2022–2023).

### Data analysis

Data analysis was conducted using Stata/IC (version 14.0). Initial assessments included item completion rates and summaries of sample composition and variations in the primary outcome (continuity of care score: NCQ subscales) by patient group at T1 and T2.

### Regression analysis

Multiple regression was used to test for differences in perceived continuity between patient groups and timepoints, controlling for potential confounding variables. Given the nesting of patients within practices and PCNs, we considered factors from patient, practice, and PCN levels that might be associated with continuity (Supplementary Table S2). To avoid exclusion of potentially significant predictors of relational continuity in the final model, a conservative *P* value of 0.2 was employed to indicate a significant association in a bivariate analysis.^31^ To test for changes in perceived continuity between timepoints, which may be owing to the introduction of PCNs, we use a multiple regression with interaction^29,30,32^ terms.


$$Continuity_{it}=\beta_{0}+\;\beta_{1}Timepoint2+\beta_{2}PatientGroup_{i}+\beta_{3}\left(Timepoint2*PatientGroup_{i}\right)+\beta_{4}X+\varepsilon_{it}$$

The interaction term for timepoints and patient group is $\beta_{3}$ . It indicates how changes in continuity from the first year to the second differs for the group compared with the reference group (younger working-age adults). Coefficients significantly different from zero indicate a change relative to the reference group. $\beta_{1}$ represents the effect of the timepoint on continuity for the reference group.

The regression models were adjusted for variables, **X**, correlated to continuity, which comprise: PCN-level (complexity, that is, size of PCN by number of practices), connectedness, practice-level (GP full-time equivalent [FTE], nurse FTE, other HCP FTE),^34,35^ and population-level (aged <5 years, >65 years)^36^ characteristics; and, from the questionnaire, HCP seen at the last appointment and how often they see their preferred GP.

## Results

The final sample size was 696, across 19 practices within five PCNs, after excluding incomplete questionnaires. The survey response rate was 23% for both T1 (*n* = 362/1547) and T2 (*n* = 350/1528). The characteristics of the study population are shown in Table 1. We also compared our findings to key characteristics and questions in the national GP Patient Survey 2022.^37^

**Table 1.** Characteristics of the study population for T1 and T2

	T1	T2	
	***n* (%**)	***n* (%**)	
**Sex**	**355**	**325**	***
Male	106 (29.9%)	139 (42.8%)	
Female	246 (69.3%)	182 (56%)	
**Age categories, years**	**351**	**314**	**
18–24	20 (5.7%)	5 (1.6%)	
25–34	44 (12.5%)	40 (12.7%)	
35–44	61 (17.4%)	48 (15.3%)	
45–54	15 (4.3%)	13 (4.1%)	
55–64	21 (6.0%)	22 (7.0%)	
65–74	86 (24.5%)	57 (18.1%)	
75– 84	75 (21.4%)	95 (30.3%)	
≥85	29 (8.3%)	34 (10.8%)	
**Age: mean (SD**)	58.8 (21.3)	62.7 (20.5)	
**Ethnicity**	**354**	**326**	***
White	288 (81.4%)	273 (83.7%)	
Non-White	66 (18.6%)	53 (16.3%)	
**Patient group**	**357**	**339**	
Adult aged >65 years with polypharmacy	185 (51.8%)	189 (55.8%)	
Adult with mild or moderate anxiety or depression	98 (27.5%)	85 (25.1%)	
Younger working-age adults	74 (20.7%)	65 (19.2%)	
**Primary care network**	**357**	**339**	
PCN A	64 (17.9%)	55 (16.2%)	
PCN B	107 (30.0%)	107 (31.6%)	
PCN C	30 (8.4%)	31 (9.1%)	
PCN D	82 (23.0%)	90 (26.6%)	
PCN E	74 (20.7%)	56 (16.5%)	

Pearson χ^2^: statistically significant differences between T1 and T2 at ***P≤0.001; **P≤0.01 levels.

At T1 69% of our sample were female and 56% at T2, 81% were of White ethnicity at T1 and 84% at T2. Fifty-two per cent were aged >65 years with polypharmacy at T1, 28% were adults with mild-to-moderate anxiety or depression, and 21% were working-age adults aged 18–45 years. They were 56%, 25%, and 19%, respectively at T2.

Results indicate some notable differences, including a higher rate of face-to-face consultations in our sample compared with the GP Patient Survey 2022^37^ data (Supplementary Table S3).

### Continuity of care scores: descriptive

Overall, adults with polypharmacy scored highest on all subscales (continuity with a preferred GP, non-preferred GP, other HCP, cooperation between HCPs within the practice and HCP cooperation cross-sector) except for continuity with an HCP outside the practice. Younger working-age adults scored lowest on all subscales. All the three patient groups reported higher continuity with a preferred GP, and lowest continuity with a non-preferred GP compared with another HCP (Table 2). Overall, continuity scores were slightly higher for T2 than T1; however, this was not statistically significant (Table 3). The slight differences observed are likely to result from increases in face-to-face appointments (from 71% at T1 to 82% at T2) and decreases in remote consultations (telephone: 67% at T1 to 55% at T2; video: 4% at T1 to 1% at T2; and email: 5% at T1 to 2% at T2) as shown in (Supplementary Table S4) where responders specified the appointment types they had.

**Table 2.** Comparison of experienced continuity with care providers, by timepoint and patient group

	**Adult aged >65 years, polypharmacy**				**Adult, anxiety or depression**				**Younger working-age adult**				
**Continuity with (care provider**)	**Timepoint 1**		**Timepoint 2**		**Timepoint 1**		**Timepoint 2**		**Timepoint 1**		**Timepoint 2**		
	*n*	**Mean (SD)**	*n*	**Mean (SD)**	*n*	**Mean (SD)**	*n*	**Mean (SD)**	*n*	**Mean (SD)**	*n*	**Mean (SD)**	
**Preferred GP**	111	3.40 (1.03)	102	3.44 (1.04)	55	3.47 (0.97)	56	3.30 (0.99)	35	3.31 (1.03)	28	3.38 (0.93)	
**Non-preferred GP**	137	2.75 (1.13)	140	2.64 (1.20)	83	2.62 (1.12)	68	2.77 (1.02)	71	2.25 (1.04)	50	2.63 (1.05)	*
**Other HCP**	130	2.92 (1.11)	137	2.97 (1.14)	58	2.83 (1.14)	50	2.95 (1.07)	61	2.45 (1.11)	42	2.86 (0.93)	*
**Cooperation between HCPs within practice**	136	3.42 (1.15)	145	3.33 (1.03)	82	3.24 (1.14)	69	3.45 (1.10)	65	3.18 (1.02)	45	3.21 (1.08)	
**HCPs outside GP practice**	115	3.27 (1.08)	122	3.31 (1.05)	52	3.30 (1.23)	48	3.38 (0.83)	49	3.10 (1.15)	39	3.19 (1.12)	
**HCP cooperation cross-sector**	96	3.46 (1.14)	107	3.40 (1.04)	45	3.20 (1.29)	39	3.51 (1.05)	43	3.04 (1.06)	38	3.32 (1.31)	

5 = higher continuity. 1 = lower continuity. Two sample *t*-test: no statistically significant difference between T1 and T2 except for perceived continuity with a non-preferred GP and other HCPs with younger working-age adults at *P≤0.05 level. HCP = healthcare professional

**Table 3.** Comparison of experienced overall continuity of care between timepoints

		**T1**		**T2**	
	***n***	**Mean (SD**)	***n***	**Mean (SD**)	**Change over time *P* value**
**Preferred GP**	201	3.40 (1.01)	186	3.39 (1.01)	0.907
**Non-preferred GP**	291	2.59 (1.12)	258	2.67 (1.13)	0.419
**Other HCP**	249	2.79 (1.13)	229	2.94 (1.09)	0.122
**Cooperation between HCPs within practice**	283	3.31 (1.12)	259	3.34 (1.06)	0.765
**HCPs outside GP practice**	216	3.24 (1.13)	209	3.31 (1.01)	0.528
**HCP cooperation cross-sector**	186	3.30 (1.17)	184	3.41 (1.10)	0.352

5 = higher continuity. 1 = lower continuity. Two sample *t*-test: no statistically significant difference between timepoints. HCP = healthcare professional

Results suggested that the more exposure responders have to certain HCPs the greater the continuity experienced.

### Patients’ experiences of continuity of care: regression

The regression results (Table 4) suggest that the polypharmacy group have consistently higher continuity of care scores compared with younger adults, after controlling for various characteristics at PCN, practice, and patient levels. In model 1, where only a limited set of variables is included, the polypharmacy group has a statistically significant association with continuity of care, with a coefficient of 0.484, which remains stable as additional covariates are added across models 2 to 4, with the coefficient only slightly decreasing.

**Table 4.** Regression table

Variables	Model 1 Coeff (95% CI) Continuity of care	Model 2 Coeff (95% CI) Continuity of care	Model 3 Coeff (95% CI) Continuity of care	Model 4 Coeff (95% CI) Continuity of care
**Younger adults (base**)	**Base**	**Base**	**Base**	**Base**
**Polypharmacy**	0.484*** [0.153 to 0.816]	0.439*** [0.183 to 0.694]	0.465** [0.092 to 0.839]	0.349** [0.037 to 0.661]
**Anxiety or depression**	0.314* [-0.055 to 0.684]	0.295 [-0.375 to 0.965]	0.283 [-0.387 to 0.952]	0.221 [-0.136 to 0.578]
**Timepoint 1 (base**)	**Base**	**Base**	**Base**	**Base**
**Timepoint 2**	0.056 [-0.376 to 0.488]	0.034 [-0.405 to 0.473]	0.011 [-0.509 to 0.532]	0.033 [-0.250 to 0.316]
**Polypharmacy # Timepoint 1 (base**)	**Base**	**Base**	**Base**	**Base**
**Polypharmacy # Timepoint 2**	-0.128 [-0.650 to 0.395]	-0.103 [-0.716 to 0.509]	-0.147 [-0.738 to 0.444]	-0.101 [-0.443 to 0.240]
**PCN characteristics**	No	Yes	Yes	Yes
**Practice characteristics**	No	No	Yes	Yes
**Patient characteristics**	No	No	No	Yes
**Constant**	2.522*** [2.262 to 2.783]	3.490*** [3.046 to 3.934]	1.180 [-1.360 to 3.720]	0.297 [-1.360 to 1.955]
***R* ^2^**	0.03	0.066	0.113	0.339
**Observations**	509	509	500	500

^*^ *P*<0.10, ^**^ *P*<0.05, ^***^ *P*<0.01. 95% confidence intervals in brackets. Differences between polypharmacy and younger adults are significant at the traditional 5% level but not 1% level. Coeff = coefficient

For patients with anxiety or depression, the results are less consistent. In the baseline model (model 1), this group shows a small positive association with continuity of care, but this loses significance once PCN characteristics are included in model 2.

An interaction term between polypharmacy status and T2 (compared with the baseline T1) was included in each model to assess any change in continuity of care over time. This interaction term was negative for all models but consistently non-significant, indicating no meaningful difference in continuity of care for polypharmacy patients between T1 and T2. Overall, these findings suggest that while reported continuity of care is higher for polypharmacy patients, this effect does not change over time.

To reduce the chance of false positives in our analysis, our sample size calculation used a significance level of 0.01 instead of the more common 0.05. Using the stricter significance level, most of our results remain the same, except that in model 1, the difference for patients with anxiety or depression compared with younger adults is no longer statistically significant.

### Factors associated with continuity of care

Regression model 2, which adjusted for PCN-level characteristics, suggests that continuity of care was positively associated with increasing connectedness and complexity (PCNs with pre-existing inter-practice collaborations), and increasing PCN size (*P*<0.01). We further controlled for practice- and patient-level characteristics (model 3). Results show that increasing PCN size is significantly associated with better continuity (*P*<0.01). Results from regression model 3 further suggest that practice rurality (*P*<0.01), and higher numbers of FTE GPs (*P*<0.1) and other HCPs (*P*<0.01) were significantly associated with increased continuity of care. Populations aged <5 years (*P*<0.01) and >65 years (*P*<0.05) were negatively associated with relational continuity.

After controlling for individual factors, regression model 4 indicates that better continuity of care was associated with increasing PCN size (*P*<0.1); when a nurse (*P*<0.05) was seen at the last appointment compared with a GP or other HCP; patients who always or sometimes saw their preferred GP (*P*<0.01), rather than those who never did; and, higher other HCP FTE (*P*<0.1). Population aged <5 years (*P*<0.1) remained negatively associated with relational continuity. Increasing PCN size remained positively associated with better continuity (*P*<0.1). Increased PCN connectivity (pre-existing inter-practice collaborations), sex, ethnicity, and increasing deprivation were not associated with any significant differences in relational continuity (see Supplementary Table S6, for details*)*

## Discussion

### Summary

This study explored adult patients’ perceptions of relational continuity in UK primary care across different age groups and medical needs and conditions. Across all patient groups, people reported better experiences of continuity when they were able to see their preferred GP. Continuity with a non-preferred GP or another HCP, was generally lower. This highlights the importance of patients having access to the HCP they know and trust. Adults with polypharmacy consistently reported the highest levels of continuity of care. This group are likely to engage with the practice frequently to arrange repeat prescriptions, vaccinations, and medication reviews. They are therefore more likely to regularly see the same HCP over time, which can maintain an ongoing and trusting relationship.

Several factors were found to influence continuity of care: (1) larger PCNs with greater collaboration between practices tended to offer better continuity of care. Practices with strong connections that worked closely together were more likely to provide patients with consistent care experiences; (2) practices with more full-time GPs and HCPs offered better continuity. This suggests that patients in these practices had more opportunities to see the same HCP regularly; (3) in some cases, seeing a nurse rather than a non-preferred GP was associated with better continuity of care. This underscores the importance of HCPs, such as nurses, in fostering strong patient–provider relationships; and (4) patients aged <5 years and >65 years were less likely to experience good continuity, which may suggest that more targeted efforts are needed to ensure consistent care for these age groups.

While there was a slight increase in continuity scores over time, it was not significant. The slight shift in how patients were receiving care, with more face-to-face appointments and fewer remote consultations may have contributed to the slight improvement in continuity scores. Lack of changes in continuity between the two time periods may be owing to PCNs employing more HCPs in different roles between these periods.

Our findings emphasise the importance of providing patients, especially those with complex health needs, such as polypharmacy, with consistent access to their preferred HCPs. Structural factors, such as PCN size, inter-practice collaboration, and staffing levels also influence patients’ experiences of continuity of care. Addressing these factors could facilitate better, more consistent care for patients.

### Strengths and limitations

The study included multiple PCNs and different patient groups, supporting broader generalisability to other healthcare settings than primary care. Second, experiences of continuity in this paper report the patient perspective, and by comparing results with the national GP Patient Survey 2022,^37^ the study was able to contextualise findings and validate them against a larger dataset. However, the absence of pre-implementation continuity data limits our ability to fully account for changes over time that are owing to PCNs, therefore any conclusions about changes should be interpreted with caution. Also, the overall survey response rate of 23% may introduce selection bias, as the sample may not fully represent the broader patient population, limiting generalisability of the findings. Notably, low response rates came from PCN C at both timepoints, compared with 30% and 32% responders from PCN B. While the regression models adjusted for several factors, they cannot account for all potential confounding variables, which could impact the robustness of the results. Additionally, our study design compares patient groups between timepoints rather than using a paired design, introducing potential confounding that may affect the reliability of conclusions about changes over time.

### Comparison with existing literature

Consultations with a usual or preferred GP are considered safer by patients.^38^ This is more so when care needs are more complex, which tends to be among older patients.^39^ In the past, evidence suggested that smaller practices offer better continuity of care,^40^ however, none specifically examines whether a larger PCN affects patients' continuity of care or access to a preferred clinician.

### Implications for research and practice

This study highlights the importance of HCPs other than GPs, particularly nurses, in providing continuity of care. This suggests that healthcare systems could empower nurses and other HCPs to build stronger relationships with patients. Patients who consult their preferred HCP report better relational continuity, therefore practices should prioritise more consistent access to a patient’s preferred GP or HCP within appointment scheduling, especially for older adults and those managing long-term conditions. The slight increase in face-to-face appointments at the second timepoint suggests a correlation between in-person interactions and continuity. Future research should explore how to best balance digital and in-person care while ensuring patients can still have face-to-face visits with their preferred HCPs. In addition, as polypharmacy patients experience better continuity of care, practices should consider targeted strategies for vulnerable populations such as older adults with complex health needs. Better continuity among larger PCNs and those with pre-existing inter-practice collaborations suggest that practices could improve integration and cooperation within their PCNs to enhance continuity for patients. The findings show if changes occurred during the study but linking them directly to PCN implementation is hard without baseline data.

## Data

The dataset relied on in this article is available from the corresponding author on reasonable request.
